# Glucocorticoid induced group 2 innate lymphoid cell overactivation exacerbates experimental colitis

**DOI:** 10.3389/fimmu.2022.863034

**Published:** 2022-08-12

**Authors:** Bingcheng Feng, Lin Lin, Lixiang Li, Xin Long, Chao Liu, Zixiao Zhao, Shiyang Li, Yanqing Li

**Affiliations:** ^1^ Department of Gastroenterology, Qilu Hospital, Cheeloo College of Medicine, Shandong University, Jinan, China; ^2^ Laboratory of Translational Gastroenterology, Qilu Hospital, Cheeloo College of Medicine, Shandong University, Jinan, China; ^3^ Advanced Medical Research Institute, Shandong University, Jinan, China

**Keywords:** inflammatory bowel disease, ulcerative colitis, HPA axis activation, innate lymphoid cell, ILC2, glucocorticoid

## Abstract

Abnormal activation of the innate and adaptive immune systems has been observed in inflammatory bowel disease (IBD) patients. Anxiety and depression increase the risk of IBD by activating the adaptive immune system. However, whether anxiety affects innate immunity and its impact on IBD severity remains elusive. This study investigated the mechanism by which anxiety contributes to IBD development in a murine model of acute wrap restraint stress (WRS). Here, we found that anxiety-induced overactivation of group 2 innate lymphoid cells (ILC2) aggravated colonic inflammation. Overactivation of the hypothalamic–pituitary–adrenal (HPA) axis is a hallmark of the physiological change of anxiety. Corticosterone (CORT), a stress hormone, is a marker of HPA axis activation and is mainly secreted by HPA activation. We hypothesized that the overproduction of CORT stimulated by anxiety exacerbated colonic inflammation due to the abnormally elevated function of ILC2. The results showed that ILC2 secreted more IL-5 and IL-13 in the WRS mice than in the control mice. Meanwhile, WRS mice experienced more body weight loss, shorter colon length, higher concentrations of IL-6 and TNF-α, more severely impaired barrier function, and more severe inflammatory cell infiltration. As expected, the serum corticosterone levels were elevated after restraint stress. Dexamethasone (DEX) was then injected to mimic HPA axis activation induced CORT secretion. DEX injection can also stimulate ILC2 to secrete more type II cytokines and exacerbate oxazolone (OXA) induced colitis. Blocking the IL-13/STAT6 signaling pathway alleviated colitis in WRS and DEX-injected mice. In conclusion, the overactivation of ILC2 induced by CORT contributed to the development of OXA-induced colitis in mice.

## Introduction

Inflammatory bowel diseases, including ulcerative colitis (UC) and Crohn’s disease (CD), are chronic and recurrent diseases that may be mediated by the immune system, genetic factors, and gut microbiota dysbiosis (1). Recurrent abdominal pain, diarrhea, hematochezia, weight loss, and fatigue are common clinical symptoms in both UC and CD, which result in a dramatic decline in the quality of life and cause a great medical burden (2, 3). Although UC and CD have similar clinical symptoms, they are completely separate in terms of pathological and immune changes. UC is localized within the colonic mucosa and submucosa, whereas CD is a chronic transmural inflammation that can involve any part of the alimentary tract (4–6). As for the immune response, CD is characterized by the overactivation of Th1 cells triggered by increased IL-18 and IL-12 production and IFNγ production (7). Unlike Crohn’s disease, type 2 cytokines such as IL-5 and IL-13 are increased in the serum and tissue of patients with UC, which may impact the treatment and prognosis of the disease (8, 9).

In addition to the adaptive immune system, innate immunity plays a role in the pathogenesis of UC (10). Innate lymphoid cells are recognized as the key modulators that maintain homeostasis and are involved in the progression of some pathological conditions. IL-22 and IL-17 released from ILC3 upregulate antimicrobial molecules such as REG3 proteins and regulate T-cell responses to commensal bacteria (11, 12). ILC3 also secretes granulocyte–macrophage colony-stimulating factor, which contributes to the progression of intestinal inflammation (13). ILC2 has been found in mucosal organs such as the intestine and lung, as well as in other tissues such as adipose and kidney, and plays a role in maintaining homeostasis (14). ILC2 expresses GATA3 and secretes type II cytokines such as IL-4, IL-5, and IL-13, which are initially found to protect against GI parasite infection by inducing eosinophil infiltration and goblet cell proliferation (15–17). Furthermore, in murine dextran sodium sulfate (DSS)-induced colitis, ILC2 secretes amphiregulin, which inhibits inflammation and promotes tissue repair and has a similar effect on influenza virus infection in the lung (18, 19). On the other hand, IL-13 secreted by ILC2 also promoted intestinal inflammation by activating STAT6 in intestinal epithelial cells, increasing gut permeability (20). As the type 2 immune response has been shown to be closely related to the pathogenesis of UC, OXA-induced colitis is considered a suitable model, and blocking the IL-25 signal of ILC2 may contribute to the remission of oxazolone-induced colitis (8, 10, 21, 22).

Anxiety and depression are recognized as risk factors for IBD and common psychiatric comorbidities in both UC and CD patients; even patients without steroid treatment or surgical intervention still show much higher psychological symptoms than the control group (23–25). Patients with anxiety or depression symptoms also have a higher risk of IBD-related surgery or hospitalization (26). However, the impact of negative mental factors on IBD pathogenesis remains unknown.

In this study, we found that excessive CORT induces aberrant activation of ILC2, secreting more type 2 cytokines and exacerbating OXA-induced colitis.

## Material and methods

### Animals and restraint stress procedure

Adult male C57BL/6J mice (6–8 weeks) were purchased from Nanjing Gempharmatech (Nanjing, Jiangsu Province, China). All animal experiments were conducted under the code of animal ethics and were approved by the Ethical Committee and Institutional Animal Care and Use Committee of Shandong University. For the acute anxiety model, anxiety was induced using a wrap-restraint stress (WRS) procedure, which was performed by placing mice into 50-ml tubes with a hole for ventilation. All immobilization operations were performed at fixed periods (10:00 am to 1:00 pm) to eliminate the effects of circadian rhythms. After immobilization, the mice were placed in their home cages.

### Intraperitoneal injection

For DEX (Selleck CAS No. 50-02-2) intraperitoneal (i.p.) injection, Dex was dissolved in dimethyl sulfoxide (DMSO, Solarbio, Beijing, China) at 10 mg/ml and injected with 5 mg/kg of vehicle (10% DMSO, 30% PEG400, 5% Tween80, 55% H_2_O) daily at 10:00 am for seven consecutive days. Mifepristone (RU486, Selleck, USA), a glucocorticoid receptor (GR) inhibitor, was injected at 20 mg/kg. RCM-1 (Selleck, USA), an IL-13/STAT6 signaling pathway inhibitor, was injected at 1.7 mg/kg. The control group was injected with the same volume of vehicle (10% DMSO, 30% PEG400, 5% Tween80, and 55% H_2_O).

### Oxazolone-induced colitis

Before the enema, the mice were anesthetized with an i.p. injection of 2% sodium pentobarbital (3.5 μl/g). Oxazolone (4-ethoxymethylene-2-phenyl-2-oxazolin-5-one) (Sigma Chemical Co., St. Louis, MO, USA) was dissolved in 50% ethanol (50% ethanol:50% H_2_O). Then 100 ul of 1% (w/v) oxazolone (OXA) was delivered *via* rectal enema, and mice that had received an enema were held in a head-down vertical position for 20 min before returning to their cages to ensure that the oxazolone was retained in the colon. Body weight and stool properties were recorded daily.

### Enzyme-linked immunosorbent assays

After the mice were euthanized, blood was collected from the heart. Untreated blood was placed on a stand for 2 h at room temperature and centrifuged at 3,000×*g* for 10 min at 4°C to obtain serum. All serum samples were put in liquid nitrogen immediately and then transferred to a −80°C freezer. Serum corticosterone levels were measured using a mouse corticosterone ELISA kit (CUSABIO, CSB-E07969m), according to the instructions of the manufacturer.

### Tissue fixation and hematoxylin and eosin staining

Colon tissue was removed and fixed in 4% paraformaldehyde for at least 24 h at room temperature and then embedded in paraffin after a standard dehydration procedure. The tissues were sectioned at 4 μm and stained with hematoxylin and eosin (HE). An objective histopathological score was determined according to leukocyte infiltration, vascular density, and epithelial cell loss (22). All the scores were summed up to evaluate the histological changes.

### qPCR

Tissue samples were ground at 4°C, and total RNA was extracted using the RNA isolater Total RNA Extraction Reagent (Vazyme, China). cDNA was synthesized from the RNA template using a ReverTra Ace qPCR RT Kit (Toyobo, Osaka, Japan). The primer sequences used for qPCR are listed in **Table 1**. PCR reactions were run in triplicate on an Applied Biosystems StepOne Real-Time PCR System (Thermo Fisher Scientific, Waltham, MA, USA). The expression of all target genes was evaluated using the 2^−ΔΔCt^ method, while GAPDH was used as an internal standard.

**Table 1:** Primer sequence for qPCR.

	Forword	Reverse
TNFa	GGTGCCTATGTCTCAGCCTCTT	GCCATAGAACTGATGAGAGGGAG
IL-6	CCTCTGGTCTTCTGGAGTACC	ACTCCTTCTGTGACTCCAGC
IL-12-p35	TCTCTATGGTCAGCGTTCCA	TTTCTCTGGCCGTCTTCAC
GAPDH	CATCACTGCCACCCAGAAGACTG	ATGCCAGTGAGCTTCCCGTTCAG

### Western blot

Total protein was extracted using RIPA lysis buffer (Beyotime, P0013B) and phenylmethanesulfonyl fluoride (Beyotime, ST506). Proteins were separated using 10% sodium dodecyl sulfate-polyacrylamide gel electrophoresis and transferred onto a PVDF membrane. The membrane was incubated with the primary antibody at 4°C overnight and then incubated with the secondary antibody for 1 h at room temperature. Immunoblots were detected using an enhanced chemiluminescent substrate (Millipore). Antibodies against ZO-1 (1:1,000; Absin, Shanghai, China), occludin (1:1,000; Absin, Shanghai, China), GAPDH (1:5,000; Proteintech, USA), and goat anti-rabbit IgG (1:5,000; Zhongshan Gold Bridge, Beijing, China) were used.

### Colonic lamina propria lymphocytes isolation and flow cytometry

Lymphocytes were extracted from the colonic lamina propria and flow cytometry was conducted as previously described (27). After removing the mesentery and adipose tissue, the colon was cut into segments (approximately 0.5 cm) and digested in complete RPMI medium (10% FBS, 1% penicillin–streptomycin) containing DNase I (150 mg/ml, Sigma) and collagenase VIII (300 U/ml, Sigma) at 37°C in a 5% CO_2_ incubator for 1.5 h. The digested intestinal segments were crushed and filtered through a 100-μm cell strainer. The interface of 80% and 40% Percoll gradients was collected after spinning at 2,500 rpm for 20 min at room temperature. Before surface staining, Fc receptors were blocked with an anti-CD16/32 antibody (eBioscience). Lymphocytes isolated from the intestinal lamina propria were stained with antibodies against the following markers: GATA3 (PB), IL-13 (Alexa Fluor 488), IL-4 (PE), IL-5 (APC), CD45.2 (AF700), CD127 (PE), CD11b (APC-Cy7), and Ly6G (FITC). Lin comprises APC-Cy7-CD3, CD5, CD19, B220, Ly6G, FcϵR1, CD11c, CD11b, Ter119, and NK1.1. For cytokine staining, cells were stimulated with 50 ng/ml phorbol myristate acetate (PMA) and 500 ng/ml ionomycin for 4 h; brefeldin A (2 μg/ml) was added 2 h before cells were harvested. Live and dead cells were distinguished using the Zombie Aqua Fixable Viability Kit (BioLegend). All flow cytometry operations were performed using a flow cytometer (Gallios, Beckman Coulter).

### Statistical analysis

Unless otherwise noted, statistical analysis was performed with the unpaired two-tailed Student’s t-test with GraphPad Prism. Results were expressed as means ± SEM. *p <0.05, **p <0.01, ***p <0.001, ****p <0.0001.

## Results

### WRS exposure exacerbates oxazolone induced colitis

**Figure 1:**
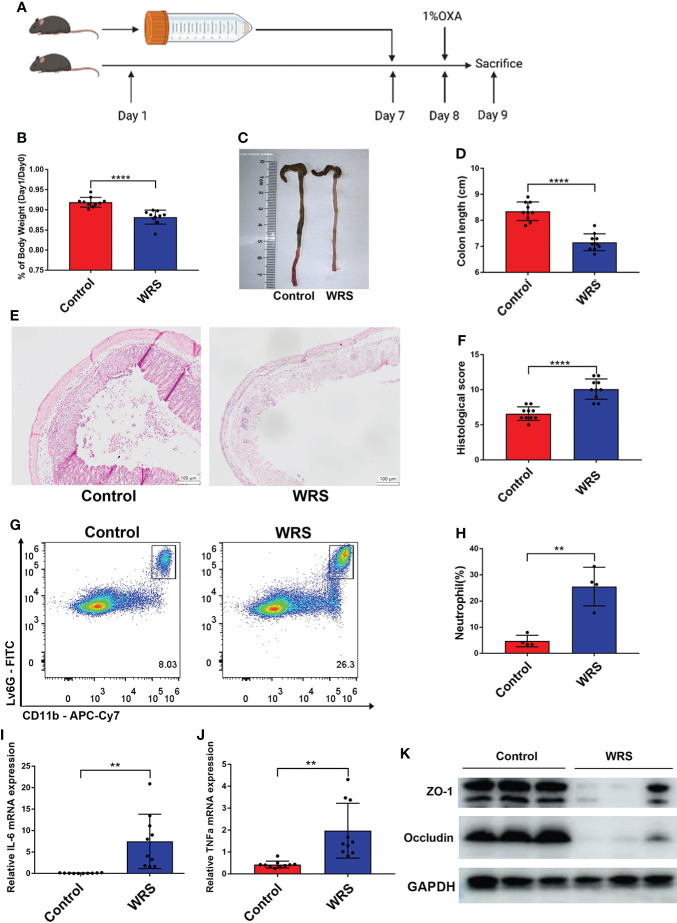
Wrap restraint stress exacerbates OXA induced colitis in mice. Mice received a 7-day WRS and then colitis was induced by 1% OXA enema. **(A)** Schematic diagram of WRS and OXA induced colitis. **(B)** Weight changes at day 1 after 1% OXA enema of WRS and control mice. **(C, D)** Colon length at day 1 after 1% OXA enema of WRS and control mice. **(E, F)** Representative HE staining [×20] and pathological score of inflamed colon tissue. **(G, H)** Flow cytometry analysis of colonic CD45+CD11b+Ly6G+ neutrophil subsets in WRS and control mice at day 1 after 1% OXA enema. **(I, J)** qPCR of Il-6 and Tnf-α mRNA of colonic tissues in WRS and control mice at day 1 after 1% OXA enema. **(K)** Immunoblot for ZO-1 and occludin in the colonic tissue of WRS and control mice at day 1 after 1% OXA enema. Data are representative of at least two independent experiments. Data are shown as mean ± SEM. **p <0.01; ****p <0.0001.

After seven days of wrap restraint stress, the mice received a 1% oxazolone enema under anesthesia (**Figure 1A**). Compared with colitis mice without WRS, mice with WRS developed more severe colitis. Both groups of mice with colitis manifested weight loss after the oxazolone challenge. However, mice with WRS lost more body weight after the oxazolone enema treatment (**Figure 1B**). Simultaneously, the colons of WRS mice were shorter than those of the control group (**Figures 1C, D**). Moreover, histological assessment and flow cytometry revealed that WRS exposure significantly amplified the severity of colitis, including architectural changes, crypt destruction, and neutrophil infiltration (**Figures 1E–H**). Furthermore, mice with WRS also expressed elevated levels of pro-inflammatory cytokines, such as IL-6 and TNF-α (**Figures 1I, J**). We also found that mice with WRS exhibited decreased expression of tight junction proteins such as ZO-1 and occludin, indicating decreased epithelial permeability (**Figure 1K**). Collectively, these data show that WRS significantly exacerbates Th-2 mediated colitis.

### Dexamethasone injection aggravates oxazolone induced colitis

**Figure 2:**
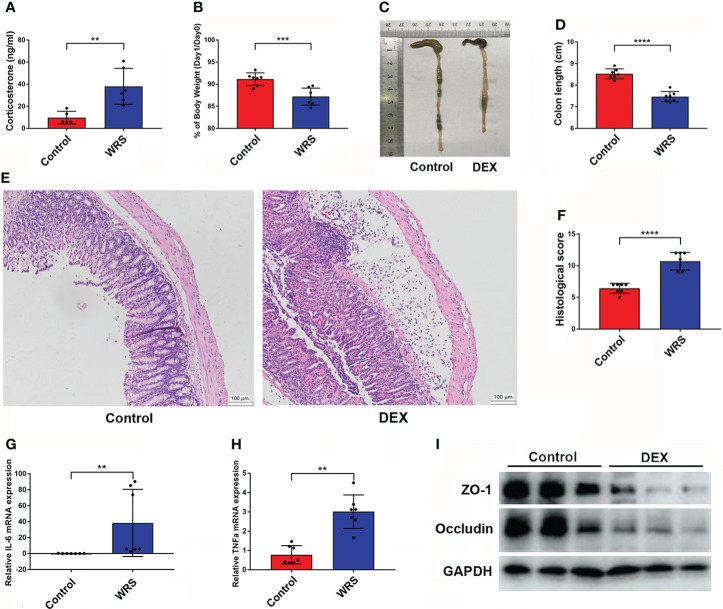
Dex injection HYPERLINK "D:/LenovoDrivers/%E6%9C%89%E9%81%93/Dict/8.9.3.0/resultui/html/index.html#/javascript:;"aggravated OXA induced colitis. DEX (5 mg/kg) or vehicle was injected for 7 consecutive days and then the mice received 1% OXA enema to induce colitis. **(A)** The serum corticosterone level of WRS and control group mice was measured by ELISA kit. **(B)** Weight changes and colon length **(C, D)** of DEX injection and control mice at day 1 after 1% OXA enema. **(E, F)** Representative HE staining of colonic sections [×20] and pathological score of inflamed colonic tissue. **(G, H)** qPCR of Il-6 and Tnf-α mRNA in colonic tissues of control and DEX injection mice at day 1 after 1% OXA enema. **(I**) Immunoblot of ZO-1 and Occludin of control and DEX injection mice at day 1 after 1% OXA enema. Data are representative of at least two independent experiments. Data are shown as mean ± SEM. **p <0.01; ***p <0.001; ****p <0.0001.

To ensure that the HPA axis was stimulated after restraint stress, after seven days of consecutive restraint, all mice were sacrificed under anesthesia, and blood was collected from the heart for serum extraction. As expected, the serum corticosterone level was increased in the WRS mice (**Figure 2A**). Therefore, we wondered whether overexpression of glucocorticoids would exacerbate OXA-induced colitis. Mice were injected with DEX (5 mg/kg) or vehicle for seven consecutive days and then received an enema of 1% oxazolone. Mice exposed to DEX showed greater body weight loss and shorter colon length (**Figures 2B–D**). HE staining showed that DEX injection promoted inflammatory cell infiltration and epithelial cell loss, and the pathological score was higher in mice injected with DEX (**Figures 2E, F**). Next, we examined inflammatory cell infiltration and the production of inflammatory cytokines using flow cytometry and qPCR, respectively. DEX treatment resulted in an increased expression of IL-6 and TNF-α (**Figures 2G, H**). The tight junction proteins occludin and ZO-1 were markedly downregulated in mice injected with DEX (**Figure 2I**). In conclusion, DEX promoted oxazolone-induced colitis, and overexpression of glucocorticoids may be responsible for WRS-related colitis aggravation.

### WRS could induce group 2 innate lymphoid cells to produce more type 2 cytokines

**Figure 3:**
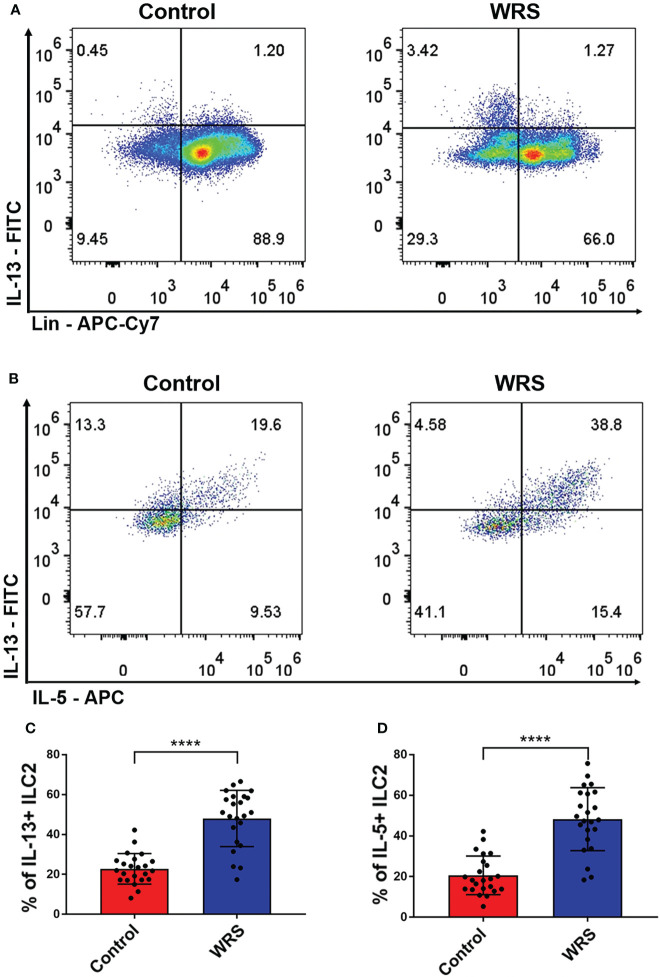
Wrap restraint stress induced overactivation of group 2 innate lymphoid cell to produce more type 2 cytokines. After 7 consecutive days restraint stress, colonic lamina propria lymphocytes were isolated from control and WRS mice; the function of ILC2 was assessed by flow cytometry. **(A)** The major cell type that secreted IL-13 in control and WRS group. **(B–D)** Flow cytometry analysis of the function of ILC2 of control and WRS mice. Data are representative of at least two independent experiments. Data are shown as mean ± SEM. ****p <0.0001.

OXA-induced colitis is a classic Th-2 mediated colitis that resembles human ulcerative colitis. As the main sources of type II cytokines were ILC2, NKT, and Th2 cells, we assessed the function of colonic lamina propria lymphoid cells using an *ex vivo* test. Lymphoid cells of colonic lamina propria were cultured in RPMI medium with PMA/ionomycin for 2 h, followed by the addition of brefeldin A for another 2 h. We found that Lin^-^GATA3^+^ cells, in other words, ILC2 produced most of the IL-5/13 in both control and WRS mice, while CD45^+^Lin^+^ cells only produced some of the IL-5/13, suggesting that ILC2 rather than Th2 or NKT cells were the major source of type II cytokines in the colonic lamina propria (**Figure 3A**). Surprisingly, we found that only Lin^-^GATA3^+^ILC2s produced considerably more IL-5/13 after restraint stress (**Figures 3B–D**). These results indicated that ILC2 secreted a major part of type II cytokines and their function was strengthened after WRS.

### Glucocorticoid could stimulate the function of ILC2s

**Figure 4:**
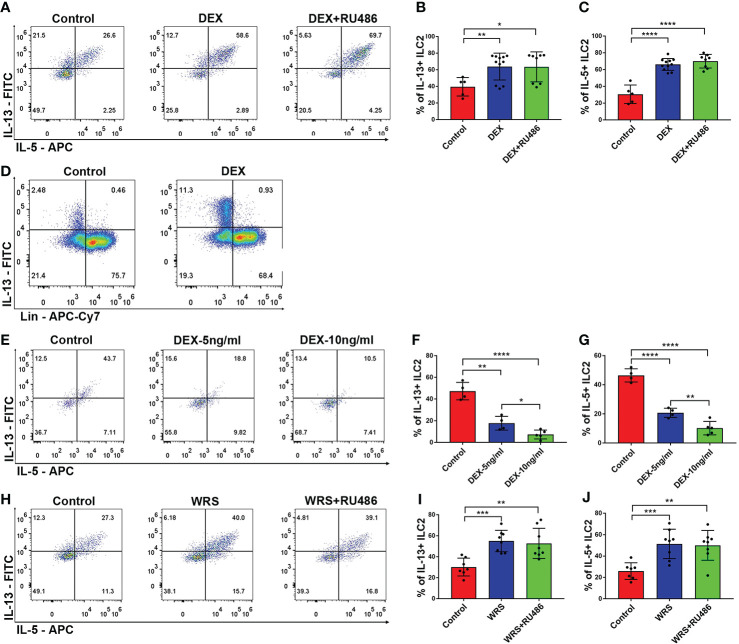
DEX injection could stimulate colonic ILC2 to secrete more IL-5/13. DEX (5 mg/kg) with or without RU486 (20 mg/kg) was injected for 7 consecutive days, colonic lamina propria lymphocytes were isolated from control and DEX injection mice, and the function of ILC2 was assessed by flow cytometry. **(A–C)** Flow cytometry analysis of IL-5 and IL-13 in colonic Lin-GATA3+ ILC2s among control, DEX injection, and RU486 injection mice. **(D)** Flow cytometry analysis of the major cell type that secreted IL-13 in control and DEX injection groups. **(E–G)** The functional changes of ILC2 under different concentrations of DEX *in vitro* tests. **(H–J)** Flow cytometry analysis of IL-5 and IL-13 in colonic Lin-GATA3+ ILC2s among control WRS and WRS + RU486 injection mice. Data are representative of at least two independent experiments. Data are shown as mean ± SEM. *p <0.05; **p <0.01; ***p <0.001; ****p < 0.0001.

To determine whether elevated levels of glucocorticoid were responsible for the stimulation of ILC2 function, the mice received an i.p. injection of DEX (5 mg/kg) with or without RU486 for seven days. After seven days of injection, we found that the function of ILC2 was significantly upregulated and CD45+Lin-GATA3+ cells were still the primary source of IL-13. This functional shift could not be eliminated by RU486 (**Figures 4A–D**). We then tested whether DEX directly influenced ILC2 using *in vitro* tests. Unlike *in vivo* studies, DEX significantly inhibited the function of ILC2 in a dose-dependent manner, and the effect was observed at a low dose (**Figures 4E–G**). Similar to the DEX group, WRS mice also received RU486 injections, and the function of ILC2 remained elevated after wrap restraint stress (**Figures 4H–J**). Glucocorticoid could stimulate the function of ILC2 to secrete more IL-5/13 indirectly, and blocking the traditional glucocorticoid receptor could not eliminate its effect on ILC2.

### Blocking IL-13/STAT6 signaling pathway could ameliorate wrap restraint stress associated colitis

**Figure 5:**
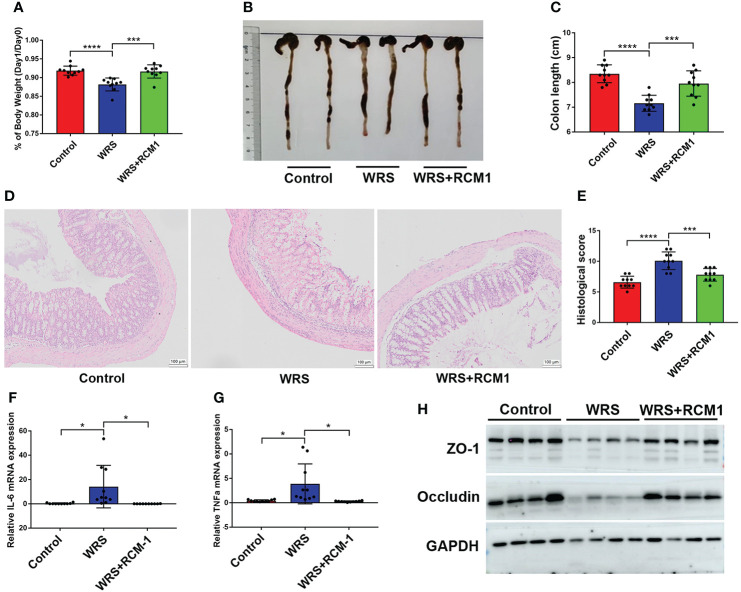
Blocking IL-13/STAT6 signaling pathway could ameliorate wrap restraint stress associated colitis. RCM-1 (1.7 mg/kg) was injected to WRS mice to block STAT6 signaling and then 1% OXA was given to induce colitis. **(A)** Body weight changes of control, WRS, WRS + RMC-1 injection mice after OXA enema. **(B, C)** Colon length at day 1 after 1% OXA enema of control, WRS, and WRS + RMC-1 injection mice. **(D, E)** Representative HE staining [×20] and pathological score of inflamed colon tissue of control, WRS, WRS + RMC-1 injection mice. **(F, G)** qPCR of Il-6 and Tnf-α mRNA in colonic tissues in control, WRS, WRS + RMC-1 injection mice at day 1 after 1% OXA enema. **(H)** Immunoblot for ZO-1 and occludin in colonic tissue in control, WRS, WRS + RMC-1 injection mice at day 1 after 1% OXA enema. Data are representative of at least two independent experiments. Data are shown as mean ± SEM. *p <0.05; ***p <0.001; ****p <0.0001.

As a typical type II cytokine, IL-13 was significantly upregulated and triggered inflammation in oxazolone-induced colitis. RCM-1 is a type of inhibitor that can block IL-13/STAT6 signaling and were administered 30 min before restraint for seven consecutive days. On the eighth day, the mice were administered an enema of 100 μl of oxazolone (1%) under anesthesia. Mice that received an RCM-1 injection developed milder colitis than mice injected with a vehicle. Mice injected with RCM-1 showed less body weight loss (**Figure 5A**), and the colons were longer (**Figures 5B, C**). Representative HE staining and the pathological score of the inflamed colon also showed that RCM-1 reduced colitis in WRS mice, with less inflammatory cell infiltration and epithelial cell loss (**Figures 5D, E**). The mRNA levels of pro-inflammatory cytokines such as IL-6 and TNF-α were also lower, and proteins that participate in intestinal tight junctions, such as ZO-1 and occludin-1, were higher than those in untreated mice (**Figures 5F–H**). These results indicate that blocking the IL-13 signaling pathway could reduce oxazolone-induced colitis and that IL-13 secreted by ILC2 may contribute to the progression of inflammation.

### OXA-induced colitis could be ameliorated by blocking IL-13/STAT6 signaling pathway in Dexamethasone injected mice

**Figure 6:**
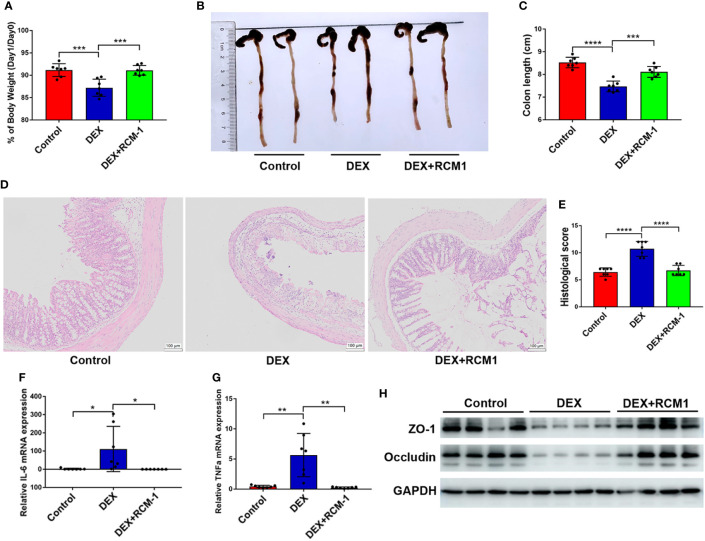
OXA-induced colitis could be ameliorated by blocking IL-13/STAT6 signaling pathway in DEX injected mice. RCM-1 (1.7 mg/kg) was injected to mice with DEX to block STAT6 signaling and then 1% OXA was given to induce colitis. **(A)** Body weight changes of control, DEX injection, DEX + RCM-1 injection mice after OXA enema. **(B, C)** Colon length at day 1 after 1% OXA enema of control, DEX injection, DEX + RCM-1 injection mice. **(D, E)** Representative HE staining [×20] and pathological score of inflamed colon tissue of control, DEX injection, DEX+RCM-1 injection mice. **(F, G)** qPCR of Il-6 and Tnf-α mRNA in colonic tissues in control, DEX injection, DEX + RCM-1 injection mice at day 1 after 1% OXA enema. **(H)** Immunoblot for ZO-1 and Occludin in colonic tissue in control, DEX injection and DEX + RCM-1 injection mice at day 1 after 1% OXA enema. Data are representative of at least two independent experiments. Data are shown as mean ± SEM. *p <0.05; **p <0.01; ***p <0.001; ****p <0.0001.

Injection of RCM-1 could significantly inhibit the pro-inflammatory influence of DEX, for mice with RCM-1 injection retained more body weight and a longer colon (**Figures 6A–C**). Histological analysis also showed that blocking IL-13/STAT6 signaling prevented the deterioration of colitis (**Figures 6D, E**). IL-6 and TNF-α levels were also lower in RU486 treated mice compared with mice that received DEX without RU486 (**Figures 6F, G**). DEX induced low expression of ZO-1 and occludin in colitis mice and was expressed more in RU486 injected mice (**Figure 6H**). Therefore, blocking the IL-13 signaling pathway could also reduce the aggravation of enteritis caused by DEX.

## Discussion

In this study, we describe and prove the hypothesis that excessive CORT could exacerbate OXA-induced colitis by stimulating ILC2 and that blocking IL-13/STAT6 signaling could reduce CORT-induced exacerbation of colitis.

Several factors, such as genetic mutations, intestinal microbiota, and environmental factors, especially social stress, have been proposed to contribute to the development of inflammatory bowel disease (28). Approximately 30% of IBD patients have anxiety/depression symptoms, and these patients tend to have more aggressive progression and poorer response to therapeutic drugs (29). Anxiety can induce systematic and local changes. Some studies have shown that anxiety increases colonic epithelial permeability by reducing the expression of tight junction-associated proteins or downregulating luminal IgA secretion (30–32). However, in other studies, anxiety did not increase epithelial permeability but aggravated colitis (33). Therefore, the specific mechanisms underlying psychological stress in IBD remain unclear. As UC is characterized by an increase in the type II immune response, OXA-induced colitis is an ideal model to investigate the influence of anxiety on colitis progression. In our study, mice that received wrap restraint stress had lower body weight and more inflammatory cell infiltration in the colon tissue after OXA enema administration, indicating that anxiety could exacerbate colitis.

Abnormal, excessive activation of the HPA axis was observed under anxiety conditions. As the terminal hormone, serum glucocorticoid level (cortisol for humans and corticosterone for mice) was used to access the activity of the HPA axis (34). Elevated cortisol levels are related to an increase in intestinal permeability (35). But whether excessive glucocorticoids contribute to the pathogenesis of IBD is still unknown. Glucocorticoids are widely used in the management of IBD for both UC and CD. Unexpectedly, Dex injection induced epithelial cell mTOR signal activation and exacerbated experimental ulcerative colitis induced by DSS (36). Therefore, abnormal glucocorticoid levels may contribute to colitis onset. In our study, DEX injection led to more severe inflammation after OXA enema administration, which was consistent with the influence of WRS. OXA-induced colitis is a classic type II immune response-mediated model; therefore, the influence of glucocorticoids on innate and adaptive lymphoid cells was investigated.

Previous studies have shown that in a restraint stress mouse model, the Th1/Th2 balance shifted toward Th2-dominant immunity (37, 38). Similar to restraint stress, glucocorticoids also showed the ability to switch the Th1/Th2 balance, as they could suppress Th1 and Th2 cell differentiation. However, Th1 cells are more affected than Th2 cells (39). One of the most accepted theories is that glucocorticoids inhibit the secretion of IL-12, which is important for Th1 cell differentiation and function (39, 40). In addition to the adaptive immune system, some studies have reported that stress can also affect innate lymphoid cells. The function of invariant natural killer T (iNKT) cells is impaired under chronic restraint stress conditions and GR is responsible for functional suppression (41).

In this study, we found that glucocorticoids had a significant influence on the function of innate lymphoid cells. ILC2 in the colonic lamina propria resulted in excessive secretion of IL-5 and IL-13 after seven days of restraint or DEX injection. ILC2 was the major source of type II cytokines, as IL-5/13 was mostly secreted by CD45+Lin−GATA3+ cells. Lin contained CD3, TCRβ, and NK1.1 to exclude Th2 and iNKT cells. Although previous studies revealed that GR mediated the influence of glucocorticoids on Th2 and iNKT cell differentiation and function (41, 42), RU486, an antagonist of GR, could not reverse restraint stress or the DEX injection-induced stimulatory function of ILC2. DEX significantly suppressed the function of ILC2 at a low concentration of 5 ng/ml *in vitro*. Therefore, the stimulation of ILC2 induced by WRS or DEX was not directly mediated by GR. Some studies have reported that IL-12 is involved in the Th1/Th2 balance (39), but the expression of IL-12 was not significantly changed in the colonic tissue of the control, WRS, and DEX groups (**Supplementary Figures 1**, **2**). RU486 could only block the classic glucocorticoid receptor (*NR3C1*), while some other receptors that could mediate the function of glucocorticoids have been discovered recently. For example, GPR97, also known as *ADGRG3*, was reported as a GPCR that binds with glucocorticoids (43), which might mediate the interaction between glucocorticoids and ILC2. Therefore, the mechanism of stress and glucocorticoid-induced activation of ILC2 is still under investigation.

IL-13 is recognized as a key cytokine that impairs epithelial barrier function by inducing apoptosis and increasing Claudin-2 expression in a dose-dependent manner (44). IL-13 secreted by ILC2 has been reported to be involved in the OXA-induced immune response, and suppression of ILC2 could alleviate inflammation (45). STAT6 is a downstream transcription factor that mediates type II immunity after IL-4/13 activation (46, 47). Increased colonic epithelial STAT6 activation has been observed in UC patients (48). IL-13 secreted by ILC2 can induce epithelial cell SATA6 activation, which affects gut permeability and exacerbates experimental colitis, and STAT6 inhibition can reverse epithelial apoptosis and claudin-2 expression (20). RCM-1 was reported to be an inhibitor that could block IL-13 and STAT6 signaling (49). As WRS- and DEX-injected mice showed overactivation of ILC2, RCM-1 was injected intraperitoneally daily for 30 min before WRS or DEX injection. As expected, RCM-1 injection reduced OXA-induced colitis in WRS- and DEX-exposed mice.

Although anti-IL-13 therapy did not achieve satisfactory goals in active UC patients (50), patients with higher IL-13 mRNA levels in the colonic mucosa were more likely to receive steroid/immunosuppressant/anti-TNF-α therapy and have a worse prognosis (8). Thus, more detailed treatment criteria should be considered to distinguish UC patients with overactivation of ILC2 in the colonic mucosa after acute stressful events who may benefit from anti-IL-13 therapy.

In conclusion, our study demonstrates that abnormal stimulation of ILC2 induced by HPA axis overactivation results in oversecretion of type II cytokines, leading to inflammation expansion in colitis. More efforts are needed to explore the effects of anxiety on the gut immune system and ILC2 may be a potential therapeutic target for colitis in patients with psychological stress.

## Data availability statement

The original contributions presented in the study are included in the article/**Supplementary Material**. Further inquiries can be directed to the corresponding authors.

## Ethics statement

The animal study was reviewed and approved by the Animal Care and Animal Experiments Committee of Shandong University (ECSBMSSDU2020-2-057).

## Author contributions

BF, SL, and YL designed the experiments. BF and LLin analyzed data and wrote the paper. XL and LLi contributed to concept and technique support. CL and ZZ supervised the project. All authors listed have made a substantial, direct, and intellectual contribution to the work and approved it for publication.

## Funding

This study was supported by the National Natural Science Foundation of China (81873550, 82070552, 81800462, and 81900486) and the Key Research and Development Program of Shandong Province (2021CXGC010506). This study was also supported by the Taishan Scholars Program of the Shandong Province.

## Conflict of interest

The authors declare that the research was conducted in the absence of any commercial or financial relationships that could be construed as a potential conflict of interest.

## Publisher’s note

All claims expressed in this article are solely those of the authors and do not necessarily represent those of their affiliated organizations, or those of the publisher, the editors and the reviewers. Any product that may be evaluated in this article, or claim that may be made by its manufacturer, is not guaranteed or endorsed by the publisher.

## Supplementary material

Supplementary Figure 1 Relative expression of IL-12 in colon tissue of WRS and control mice.

Supplementary Figure 2 Relative expression of IL-12 in colon tissue of DEX injection and control mice.

Supplementary Figure 3 Gating methods of ILC2.
