# Supplementary figures and images for: Glucocorticoid induced group 2 innate lymphoid cell overactivation exacerbates experimental colitis
Source: Front Immunol. 2022 Aug 12;13:863034. doi: 10.3389/fimmu.2022.863034 (PMC9411106; doi:10.3389/fimmu.2022.863034)

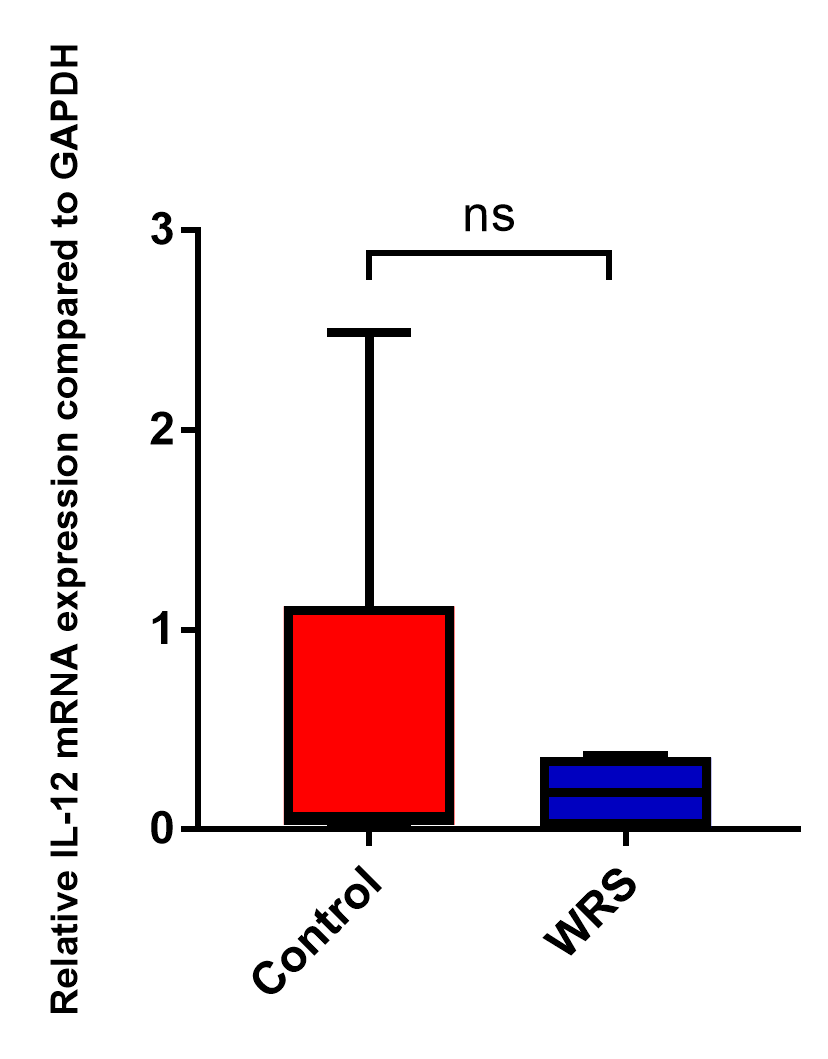

Supplement: Supplementary Figure 1 — Relative expression of IL-12 in colon tissue of WRS and control mice. [file DataSheet_1.zip › Supplementary Figure/Supplementary Figure 1.tif]

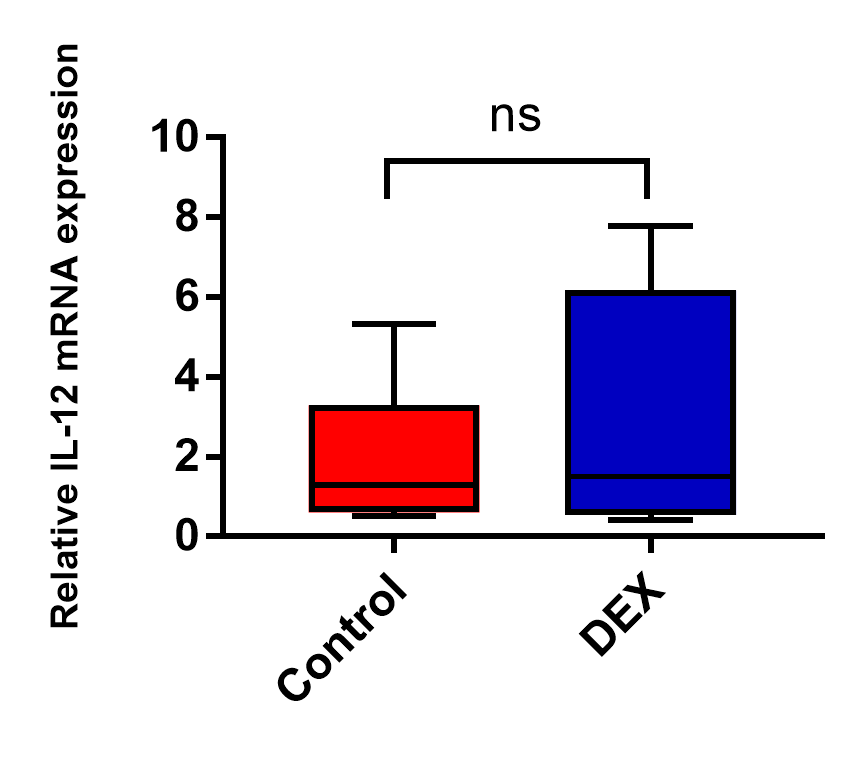

Supplement: Supplementary Figure 1 — Relative expression of IL-12 in colon tissue of WRS and control mice. [file DataSheet_1.zip › Supplementary Figure/Supplementary Figure 2.tif]

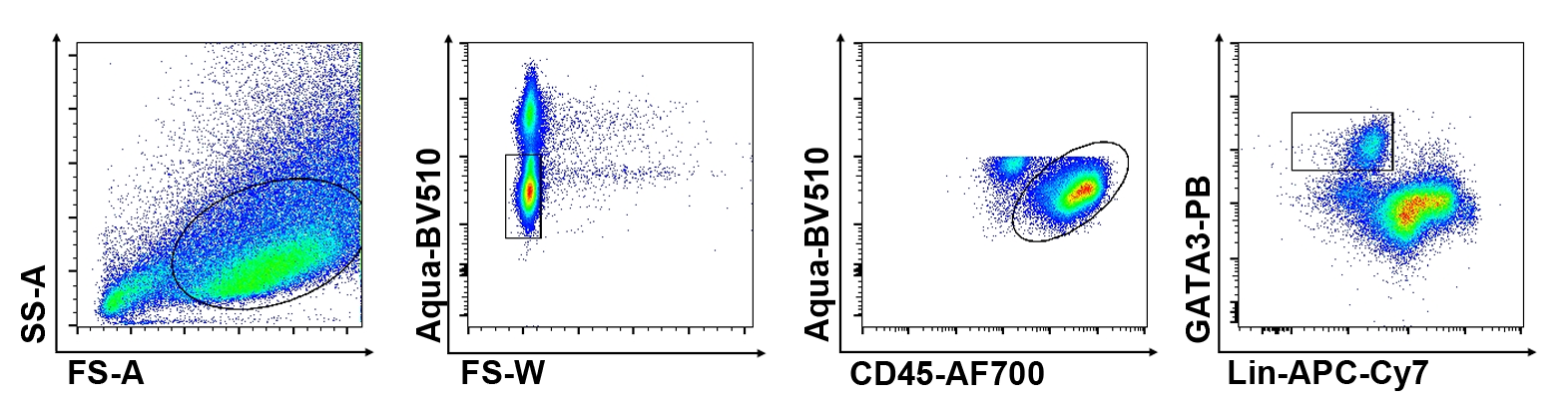

Supplement: Supplementary Figure 1 — Relative expression of IL-12 in colon tissue of WRS and control mice. [file DataSheet_1.zip › Supplementary Figure/Supplementary Figure3.tif]
